# Rational engineering of *Streptomyces albus* J1074 for the overexpression of secondary metabolite gene clusters

**DOI:** 10.1186/s12934-018-0874-2

**Published:** 2018-02-17

**Authors:** Dimitris Kallifidas, Guangde Jiang, Yousong Ding, Hendrik Luesch

**Affiliations:** 1 Department of Medicinal Chemistry, College of Pharmacy, University of Florida, Gainesville, FL 32610 USA; 2 Center for Natural Products, Drug Discovery and Development (CNPD3), University of Florida, Gainesville, FL 32610 USA

**Keywords:** Secondary metabolism, *Streptomyces albus* host engineering, Heterologous expression, Synthetic biology

## Abstract

**Background:**

Genome sequencing revealed that *Streptomyces* sp. can dedicate up to ~ 10% of their genomes for the biosynthesis of bioactive secondary metabolites. However, the majority of these biosynthetic gene clusters are only weakly expressed or not at all. Indeed, the biosynthesis of natural products is highly regulated through integrating multiple nutritional and environmental signals perceived by pleiotropic and pathway-specific transcriptional regulators. Although pathway-specific refactoring has been a proved, productive approach for the activation of individual gene clusters, the construction of a global super host strain by targeting pleiotropic-specific genes for the expression of multiple diverse gene clusters is an attractive approach.

**Results:**

*Streptomyces albus* J1074 is a gifted heterologous host. To further improve its secondary metabolite expression capability, we rationally engineered the host by targeting genes affecting NADPH availability, precursor flux, cell growth and biosynthetic gene transcriptional activation. These studies led to the activation of the native paulomycin pathway in engineered *S. albus* strains and importantly the upregulated expression of the heterologous actinorhodin gene cluster.

**Conclusions:**

Rational engineering of *Streptomyces albus* J1074 yielded a series of mutants with improved capabilities for native and heterologous expression of secondary metabolite gene clusters.

**Electronic supplementary material:**

The online version of this article (10.1186/s12934-018-0874-2) contains supplementary material, which is available to authorized users.

## Background

Talented bacteria, characterized by tens of secondary metabolite gene clusters, dedicate ~ 10% of their genomes to the corresponding biosynthetic functions [1]. The continuously reducing cost of genome sequencing renders the detection of diverse biosynthetic gene clusters rather a routine in a natural product research lab. What still hampers the large scale discovery and characterization of new bioactive molecules, however, is the expression of these gene clusters either in native or heterologous hosts. The biosynthesis of natural products is highly regulated and gene clusters often remain silent until suitable conditions are met. The expression of secondary metabolite pathways is under the control of tight and complex hierarchical regulatory networks that integrate multiple nutritional and environmental signals perceived by pleiotropic and/or cluster-situated transcriptional regulators (CSRs) [2]. Although pathway-specific refactoring and CSR-engineering has been proved to be a productive approach for the activation of individual gene clusters [3, 4], the need for generation of orthogonal sets of promoters and repressor sites can be laborious and challenging. A more broadly applicable approach would be more desirable where pleiotropic regulators affecting more than one biosynthetic pathway are targeted to amplify their positive signals in favor of secondary metabolism.

*Streptomyces albus* J1074 is widely used for heterologous expression studies due to its minimized genome (6.8 Mb) that allows fast growth [5]. The strain has been used to express diverse gene clusters and proved to be a preferred host for expression of metagenomic DNA clones encoding secondary metabolites [6–10]. Importantly, organic extracts from routine laboratory *S. albus* fermentation broths lack any endogenous secondary metabolites. Therefore, *S. albus* J1074 is an ideal strain for further optimization of its gene expression capabilities by targeted genetic engineering. Development of genetic engineering protocols for the activation of native gene clusters could also be useful for heterologous expression of foreign gene clusters in the same engineered host. Previously, a number of *S. albus* native metabolites have been characterized by targeted engineering of their corresponding cryptic gene clusters [11]. Not much effort, however, has been dedicated to the awakening of these metabolic pathways by modulating global regulators, which may have broader applicability for the heterologous expression of diverse biosynthetic gene clusters using *S. albus* as a host.

With the broad heterologous expression efficacy in mind, *S. coelicolor* M145 derivative strains have been previously constructed where endogenous gene clusters have been deleted to alleviate precursor competition and mutations to ribosomal proteins and RNA polymerase have been incorporated for higher transcriptional and translational fidelity [12]. On the other hand, pleiotropic regulators can be targeted to optimize the expression of natural products. Many global regulators have been identified in *S. coelicolor*, a model strain for genetic studies, and in the industrially important strains *S. avermetilis* and *S. griseus* (Table 1) [13–36]. For example, phosphofructokinase (*pfk*) has been shown to link primary and secondary metabolisms. The observed antibiotic downregulatory role of wblA may also be a secondary result of its direct role in the primary metabolism. Other pleiotropic DNA binding regulators like cAMP receptor protein (CRP) can recognize cognate binding sites in multiple gene clusters in response to not yet identified signals or, as in the case of DasR, can modulate their binding activity in response to glycosylated sugars. Additional global regulators can induce secondary metabolism in response to nutrient starvation and phosphate limitation.

**Table 1:** List of well-studied global regulators in *Streptomyces*

Global regulator	Role	Effect on secondary metabolism	References
WblA*	Antibiotic downregulator	Negative	[13–15]
*AdpA*	Central transcriptional regulator; AdpA represses the transcription of wblA in *S. coelicolor*	Positive	[16]
*DasR*	Regulator of secondary metabolite gene expression in response to phosphorylated amino sugars	Negative	[17]
*AtrA*	Transcriptional activator of actinorhodin; antagonist to DasR	Positive	[18, 19]
Pfk*	Phosphofructokinase; key enzyme in glycolysis that controls metabolic fluxes affecting secondary metabolism	Negative	[20, 21]
*KbpA*-*AfsKRS*	Gene cascade linking phosphate and secondary metabolisms	Positive	[22]
*PhoR*-*PhoP*	Two component system regulating phosphate assimilation; overlaps with AfsKRS regulon	Negative	[23, 24]
*AfsA*-*ArpA*	Genes required for the biosynthesis and function of γ-butyrolactone A-factor in *S. griseus*	Positive	[25, 26]
CRP*	cAMP receptor protein; activates transcription of biosynthetic genes and controlling production of precursors; partially shared regulon with that of AfsKRS and PhoRP	Positive	[27]
*AbsA1/2*	Two component system controlling antibiotic biosynthesis in *S. coelicolor*	Negative	[28]
*AfsQ1/2*	Two component system controlling antibiotic biosynthesis in *S. lividans*	Positive	[29]
*RelA*	ppGpp synthetase gene; stringent response-induced antibiotic production	Positive	[30, 31]
*RpoB*	RNA polymerase subunit; mutations conferring rifampicin resistance and mimicking stringent response-induced secondary metabolism activation	Positive	[32]
*RpsL*	Encodes for S12 ribosomal protein; mutations conferring resistance to streptomycin promote secondary metabolism activation	Positive	[33]
*BldA*	Encodes the tRNA for the rare leucine TTA codon found in many secondary metabolite pathway-specific regulators	Positive	[34, 35]
SCO1712*	Antibiotic downregulator found *in S. coelicolor*	Negative	[36]

Gene homologues have been found in many *Streptomyces* strains but not necessarily with the same impact on secondary metabolism

The *S. albus* gene homologues that are targeted in this study are indicated with an asterisk

We aimed to generate a series of engineered *S. albus* strains harboring multiple targeted pleiotropic gene modifications that would enhance secondary metabolite production. The selection of the candidate global regulators to be modified was based on the following criteria. (1) They should affect secondary metabolite overproduction in diverse ways (e.g., increased precursor supply and biosynthetic gene transcriptional activation); (2) they should not be members of the same regulon; (3) a candidate regulator should act consistently either as activator or repressor of secondary metabolism in most *Streptomyces* strains studied. For strain improvement purposes, gene deletions of protein repressors are preferred from overexpression of activators because there is no need to maintain multiple selection markers during genetic engineering experiments.

In this study we deleted *pfk* and *wblA* homologues in the *S. albus* J1074 genome yielding single and double mutants. The resulting mutant strains were further engineered by the introduction of the *crp* gene from *S. coelicolor* under the control of strong constitutive ermE* promoter to assess the potential cumulative effects towards the expression of native and heterologous gene clusters.

## Methods

### Bacterial strains and media used

Bacterial strains used in this work were *S. albus* J1074 and derivative mutants constructed in this study. *Escherichia coli* EPI300 (Epicentre) and S17.1 strains were used for subcloning and intergeneric conjugation, respectively. Growth medium for *S. albus* was tryptone soy broth (TSB) for genomic DNA isolation, and mannitol-soy flour agar (MS) was used for sporulation and R5A as regular production medium. LB medium was used for routine *E. coli* growth. When plasmid-containing clones were grown, media were supplemented with appropriate antibiotics: ampicillin (100 µg/ml), hygromycin (100 µg/ml), apramycin (50 µg/ml), chloramphenicol (12.5 µg/ml), when required.

### *S. albus* J1074 genomic DNA isolation and fosmid DNA library construction

Genomic DNA was isolated from *S. albus* mycelia collected from 2-day cultures grown in TSB. The mycelia pellet was lysed with lysozyme solution (0.5 M sucrose, 25 mM Tris–HCl, 5 mM EDTA and 2 mg/ml lysozyme) at 37 °C for 30 min. EDTA and SDS were added to 50 mM and 0.5% (final concentration) respectively. After thorough mixing, 1/3 volume of phenol/chloroform was added and mixed to emulsify. The mixture was spun at 10,000 rpm for 10 min at 4 °C and DNA was precipitated from the aqueous phase with 0.7 vol isopropanol in the presence of 1/10 vol sodium acetate, pH 5.5. The DNA pellet was washed with 70% ethanol, air-dried and resuspended in TE buffer. Fosmid DNA library was constructed using the CopyControl HTP fosmid production kit (Epicentre) following the manufacturer’s instructions.

### In-frame phosphofructokinase *pfk*_*SA*_ gene (XNR_1407) deletion

**Fig. 1:**
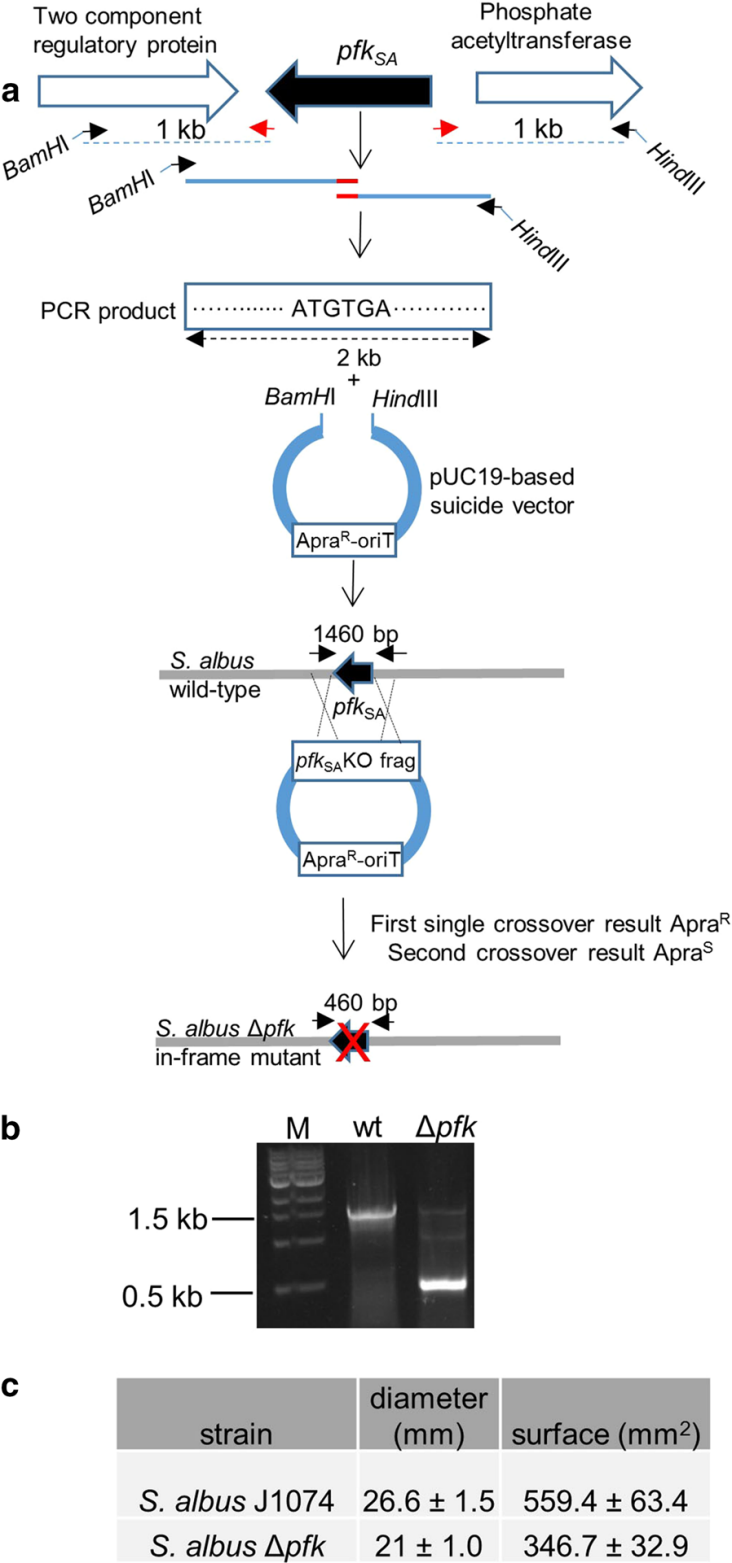
**a** Strategy for in-frame deletion of *pfk* gene in *S. albus*. **b** PCR confirmation of *pfk* deletion; M, 1 kb ladder. Solid black arrows represent primers used for PCR screening and sizes of the PCR products are indicated. **c** Comparison of sensitivity to diamide (100 mM) between *S. albus* J1074 and the Δ*pfk* derivative strain using diamide disc assays. The table shows the diameter and area of the halo formed around a disk impregnated with diamide. Values are means of three replicates. ± standard deviation (p < 0.01). Statistical significance was calculated with Student’s t-test

A suicide vector for *Streptomyces* gene deletions through homologous recombination was constructed based on pUC19 (New England Biolabs) (Fig. 1). The vector was digested with *Sca*I and *Eco*RI and ligated with *Dra*I–*Eco*RI fragment from pOJ436 carrying the oriT-apramycin cassette. Primers ALpfk1 5′-ATCGGGATCCTGGTCGACAACGCGATGGAGG-3 and ALpfk2 5′-AGCAGGAGAGACAGCACG*ATGTGA*ACCGGCTCCGCGCACACG-3′ were used to amplify 1 kb flanking region downstream of *pfk* gene. Primers ALpfk3 5′-CGTGTGCGCGGAGCCGGT*TCACAT*CGTGCTGTCTCTCCTGCT-3′ and ALpfk4 5′-ATCGAAGCTTGCCCAGCAGAACCGTTCCGTC-3′ were used to amplify 1 kb flanking region upstream of *pfk* gene. Engineered restriction sites are underlined in the primer sequences and the start/stop codon fusion site is in italics. Standard 20 µl PCR reaction mix contained 1× G buffer (Epicentre), 50 pmol of each primer, 2.5 U*Taq* polymerase (New England BioLabs), and 100 ng gDNA. A 2-step PCR protocol was used with the following conditions: 1 cycle at 95 °C followed by 30 cycles consisting of 40 s at 95 °C and 3 min at 72 °C, followed by a final extension at 72 °C for 5 min. The two PCR products were gel-purified and used as a template for overlapping PCR (same protocol as before) with primers ALpfk1 and 4 to generate a 2 kb fragment. The PCR product was gel-purified and digested with *BamH*I and *Hind*III and ligated to the described suicide vector that has been similarly digested. *E. coli* S17.1 strain was transformed with the resulting plasmid and used for conjugal transfer into *S. albus* using published protocols [37]. Apramycin resistant colonies carrying single crossover were streaked on MS agar plates with no selection for sporulation. Spores were diluted to yield single colonies and spread on MS agar plates. Double crossover mutants were identified by replica plating using Difco nutrient agar (DNA) plates with/out apramycin selection (10 µg/ml). Correct deletion of the target gene in the mutant chromosome was further verified via PCR amplification using primers ALpfkconfF 5′-GAGGTCGGCATCTCCCGCATC-3′ and ALpfkconfR 5′-ACTCCGACGATACCGGTGCG-3′. PCR reaction mix was the same as before and PCR protocol was 1 cycle at 95 °C followed by 30 cycles consisting of 40 s at 95 °C, 40 s at 58 °C and 40 s at 72 °C, followed by a final extension at 72 °C for 5 min.

### In-frame *wblA*_*SA*_ gene (XNR_2735) deletion

**Fig. 2:**
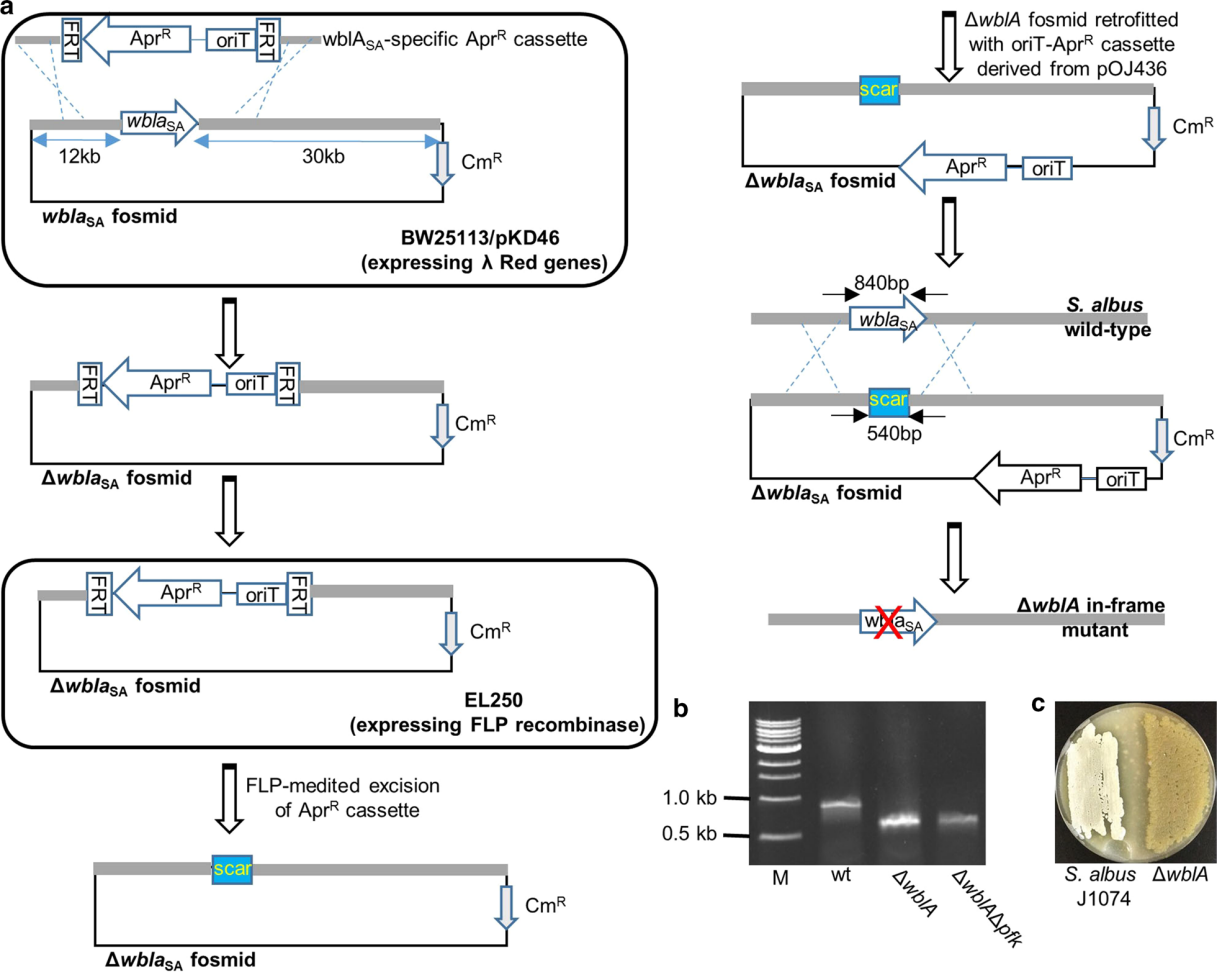
**a** Strategy for in-frame deletion of the *wblA* gene in *S. albus* using λ-mediated recombineering. **b** PCR confirmation of unmarked *wblA* deletion in different backgrounds. M, 1 kb ladder. Solid black arrows represent primers used for PCR screening and sizes of the PCR products are indicated. **c** *S. albus* Δ*wblA* mutant showed a sporulation-deficient phenotype on MS agar media

The *S. albus* fosmid library was screened by PCR using primers ALwblAF 5′-CCATCGGCACGTACCTGGCC-3′ and ALwblAR 5′-ATGTCCTTCCTGTCCCCGGC-3′. A single fosmid containing the full-length *wblA*_SA_ gene was recovered by PCR screening of serially diluted, PCR-positive library pools. PCR reaction mix contained 1× G buffer (Epicentre), 50 pmol of each primer, 2.5 U*Taq* polymerase (New England BioLabs) and 1 µl of the corresponding library pool and the PCR protocol was 1 cycle at 95 °C followed by 30 cycles consisting of 40 s at 95 °C, 40 s at 57 °C and 40 s at 72 °C, followed by a final extension at 72 °C for 5 min. The *wblA* ortholog was deleted using λ-mediated recombineering approach (Fig. 2). The *wblA*_SA_-specific *aac(3)IV*-*oriT* resistance cassette flanking by two FRT sites was amplified from pIJ773 using primers SAwblAredF 5′-TGGGGGAGCCTCGATTCGGGAGAGGACGGCGCCGGTATGATTCCGGGGATCCGTCGACC-3′ and SawblAredR 5′-GGTTCCCGTACTCCTCGCTCGCCCTTGCCGGCCGGTCTATGTAGGCTGGAGCTGCTTC-3′. The amplified cassette was transformed into *E. coli* BW25113/pKD46 containing the recovered *wblA*_SA_-containing fosmid and transformants were selected on apramycin and chloramphenicol LB agar plates. Gene replacement was confirmed by PCR analysis of the mutated (Δ*wblA*_SA_) fosmid using ALwblAF/R primers. To generate seamless gene deletion, the mutated fosmid was transformed into *E. coli* EL250 strain expressing FLP recombinase that catalyzes the recombination between the FRT sites. Following induction with l-arabinose, the excision of the apramycin resistant cassette was detected by patching single colonies on LB agar plates with/out 50 µg/ml apramycin. In-frame deletion mutants were verified by PCR using ALwblAF/R primers as before. The confirmed mutated fosmid was retrofitted with oriT-apramycin cassette by λ-mediated recombineering using primers pCCFRedF 5′-GTAACCTCGGTGTGCGGTTGTATGCCTGCTGTGGATTGCCGCAACGTTGTTGCCATTGC-3′ and pCCFRedR 5′-AGCGATGAGCTCGGACTTCCATTGTTCATTCCACGGACAAATCCCCGATCCGCTCCACG-3′. The cassette was amplified from pOJ436. The pCCF2 cloning vector sites targeted for recombination are underlined in the primer sequences. The final retrofitted and mutated *wblA*_SA_-containing fosmid was introduced into *E. coli* S17.1 cells for conjugation into *S. albus*. Double crossover mutants were confirmed by PCR using ALwblAF/R primers.

### Overexpression of *crp*_SC_ gene from *S. coelicolor* M145 (SCO3571) in *S. albus* J1074

A *crp* overexpression plasmid was made by cloning the *crp*_SC_ gene and its downstream sequence immediately downstream of the ermE* promoter in the pIJ10257 vector. The *crp* coding sequence was PCR amplified from *S. coelicolor* M145 gDNA using primers CrpF 5′-GAGAACTCATATGGACGACGTTC-3′ and CrpR 5′-CGTAAGCTTGGCCTAGGTCGCAGGGAC-3′. Engineered *Nde*I and *Hind*III sites in the forward and reverse primer, respectively, is underlined. PCR cycling conditions were 1 cycle at 95 °C followed by 30 cycles consisting of 40 s at 95 °C, 40 s at 58 °C and 40 s at 72 °C, followed by a final extension at 72 °C for 5 min. PCR product was gel-purified, digested with *Nde*I/*Hind*III and ligated to similarly digested pIJ10257 plasmid. Recombinant plasmid was introduced into *E. coli* S17.1 cells for conjugation into *S. albus*. Exconjugants were selected on MS agar plates supplemented with 25 µg/ml nalidixic acid and 50 µg/ml hygromycin.

### Knock-out of paulomycin gene cluster

For the isolation of paulomycin gene cluster, the *S. albus* fosmid library was screened by PCR using primers pml10F 5′-GGGATTCCCTGAGCGGAGTAC-3′ and pml10R 5′-GGTTTCCAGGGGCCCTTCTAG-3′. A single fosmid containing *pml*1–*pml*19 genes (entire gene cluster contains 42 genes) was recovered by PCR screening of serially diluted, PCR-positive library pools. PCR conditions were the same as used for wblA_SA_—containing fosmid isolation. The recovered *plm* fosmid was digested with *Xho*I restriction enzyme and subsequently self-ligated to eliminate 13 genes out of 19 cloned *pml* genes including pml10 pathway-specific regulator required for the transcriptional activation of the gene cluster (Fig. 5). The resulting minimized *plm* fosmid was retrofitted with oriT-apramycin cassette by recombineering as before and introduced into *E. coli* S17.1 cells for conjugation into *S. albus* and derivative mutants. Double crossover mutants were confirmed by PCR using ΔpaulconfF 5′-GAAACCGCTCCGTCCGTCCGACACC-3′ and ΔpaulconfR 5′-TGCATCCGCAGCACCAGCAGG-3′ primers. PCR conditions were 1 cycle at 95 °C followed by 30 cycles consisting of 40 s at 95 °C, 40 s at 60 °C and 40 s at 72 °C, followed by a final extension at 72 °C for 5 min.

### Cloning and site-specific integration of actinorhodin gene cluster into *S. albus* J1074 and derivative strains

For the isolation of actinorhodin gene cluster, the *S. coelicolor* fosmid library was screened by PCR using primers Act85F 5′-CTTAAATCCTCGAAGGCGAC-3′ and Act85R 5′-GCGCCCATCAGTTTGGCGTG-3′. PCR conditions were 1 cycle at 95 °C followed by 30 cycles consisting of 40 s at 95 °C, 40 s at 55 °C and 40 s at 72 °C, followed by a final extension at 72 °C for 5 min. Four PCR-positive single clones were recovered. Two clones contained partial actinorhodin gene cluster and the other two harbored actinorhodin gene cluster with different sizes of flanking regions. The fosmid with the largest DNA sequence flanking the entire actinorhodin gene cluster (SCO5067–SCO5104) was subsequently retrofitted with oriT-Apra^R^ cassette. For that, pOJ436 plasmid was double digested with *Pml*I–*Sma*I and 1.8 kb fragment containing the cassette was gel-purified and ligated with *Psi*I-digested and dephosphorylated actinorhodin-containing fosmid. Correct recombinant fosmid was confirmed by PCR using primers Act85F/R as above and used to transform *E. coli* S17.1 cells for conjugation into *S. albus* strains. Blue-pigmented exconjugants were easily selected on MS agar plates supplemented with 25 µg/ml nalidixic acid and 50 µg/ml apramycin and verified for the actinorhodin integration by PCR.

### Actinorhodin production and antimicrobial assays

Three biological replicates were tested using three confirmed colonies from each conjugation of actinorhodin cluster into *S. albus* J1074, *S. albus*+pIJ10257ermE*crp and *S. albus Δpfk*+pIJ10257ermE*crp. These colonies were streaked on MS agar plates to yield fully confluent spore lawns. Following 6 days of incubation at 30 °C, the agar from each plate was cut into small pieces and immersed into 50 ml of 1 M KOH. The tubes were left overnight at 4 °C with agitation. The samples were then spun at 4000×*g* for 10 min and the absorbance of the supernatant was measured at 640 nm. Actinorhodin concentration was calculated according to the Lambert–Beer’s law using molar extinction coefficient of 25,320/M/cm that corresponds to pure actinorhodin [38].

*Bacillus cereus* overnight cultures grown in LB were diluted by 10^6^-fold. Aliquots (100 μl) of the diluted culture were added to individual wells of a 96-well plate starting from the second column. Diluted culture (195 µl) were then added to the wells of the first column. Crude organic extracts were resuspended in methanol at 20 mg/ml. These solutions (5 µl) were added to the wells of the first column in the microtiter plate and then serially diluted twofold per well across the plate. The plates were incubated at 30 °C for 18–24 h. Concentration of 500, 250, 125, 62.5, 31.25, 15.6, 7.8, 3.9, 1.95, 0.97, 0.48, 0.24 μg/ml were tested for each crude extract. The final methanol concentration was kept at 2.5%. Minimum inhibitor concentrations are reported as the lowest concentration at which no bacterial growth was observed.

### Diamide sensitivity assays

Lawns of *S. albus* J1074 wild-type and Δ*pfk* mutant were generated by overlaying R5A plates (sucrose 100 g/l, K_2_SO_4_ 0.25 g/l, MgCl_2_ 10.12 g/l, glucose 10 g/l, casamino acids 0.1 g/l, yeast extract 5 g/l, MOPS 21 g/l, NAOH 2 g/l, R2YE trace elements 2 ml/l, 15 g/l agar) with 3 ml soft Nutrient Agar containing 10^7^ fresh spores. Immediately after plating, paper discs soaked in 100 mM diamide were added and plates were incubated at 30 °C for 24 h.

### Crude extract production for screening

Crude extracts for screening purposes were generated from 50-ml cultures of *S. albus* strains and derivative mutants grown in R5A liquid media. After 6 days of growth, cultures were extracted twice with an equal volume of ethyl acetate and the dried extracts were then used for screening.

### LC–MS profiling of engineered *S. albus* secondary metabolite content

The crude extracts were dissolved in LC–MS grade methanol and centrifuged for 30 min. The resulting clear supernatant (10 μl) was used for LC–MS analysis. A SHIMADZU Prominence UPLC system fitted with an Agilent Poroshell 120 EC-C18 column (2.7 μm, 4.6 × 50 mm) coupled with a Linear Ion Trap Quadrupole LC/MS/MS Mass Spectrometer system was used in the studies. Acetonitrile (B)/water (A) containing 0.1% formic acid were used as mobile phases with a linear gradient program (10–99% solvent B over 40 min) to separate chemicals by the above reverse phase HPLC column. The column at 30 °C was eluted first with 10% solvent B (acetonitrile with 0.1% formic acid) for 3 min and then with a linear gradient of 10–50% solvent B in 15 min, followed by another linear gradient of 50–99% solvent B in 12 min. After eluting in 99% solvent B for 5 min, the linear gradient of 99–10% solvent B in 1 min was used. The column was further re-equilibrated with 10% solvent B for 4 min. The flow rate was set as 0.5 ml/min, and the products were detected by a PDA detector. For MS detection, the turbo spray conditions included curtain gas: 30 psi; ion spray voltage: 5500 V; temperature: 600 °C; ion source gas 1:50 psi; ion source gas 2:60 psi. For MS/MS analysis, the collision energy was 12 eV.

## Results

### Construction of *S. albus* engineered strains

**Fig. 3:**
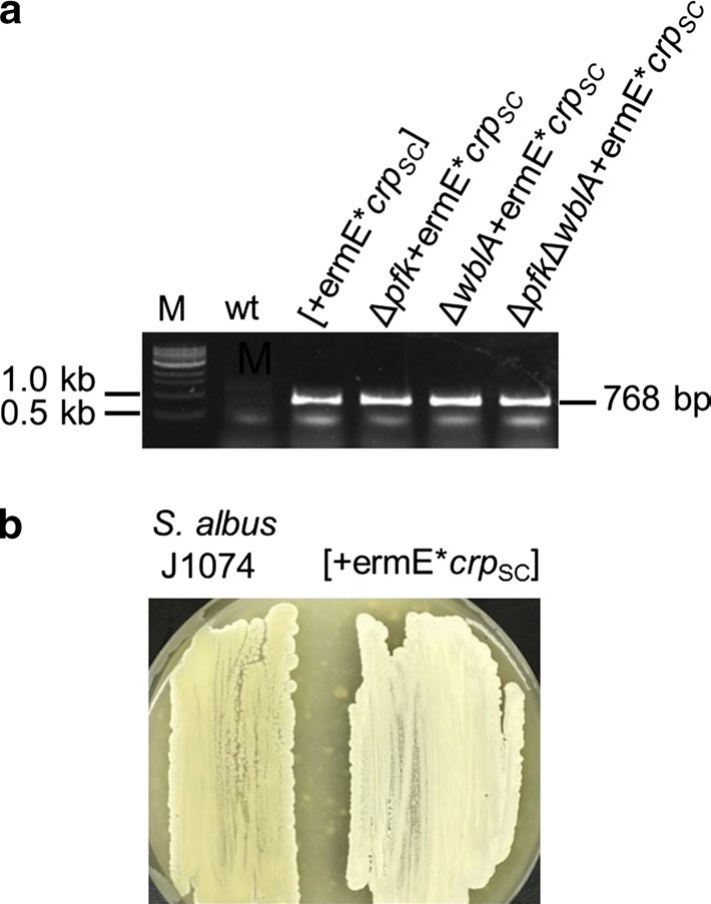
**a** PCR confirmation of pIJ10257-ermE*crp_SC_ plasmid integration into various *S. albus* backgrounds. For the PCR screening, primers were used to amplify the coding region of *crp*_SC_ gene. The expected size of the PCR product was 768 bp. M, 1 kb ladder. **b** Phenotypes of *S. albus* J1074 *vs S. albus*+erm*crp_SC_ cultured on MS solid media

**Fig. 4:**
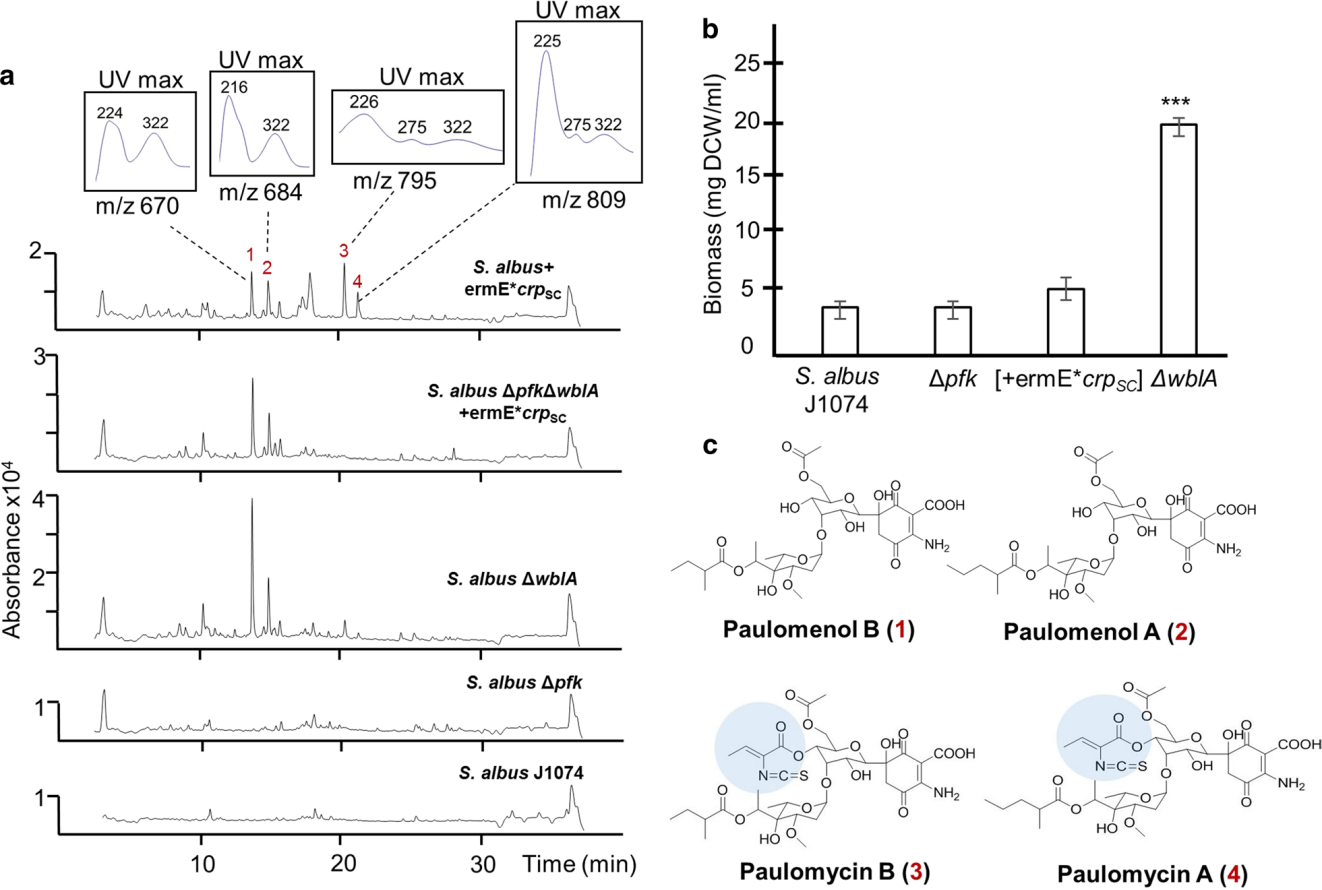
**a** LC–MS analysis of ethyl acetate extracts derived from 50 ml R5A cultures inoculated with wild-type and *S. albus* mutant strains. Cultures were grown for 6 days at 30°C. The major peaks that are present in Δ*wbla* and [+ermE*crp_SC_] backgrounds but absent in wild-type and Δ*pfk* strains are identified as paulomycin/paulomenol molecules. Corresponding masses (M + Na^+^) and maximum UV absorption spectra are shown. **b** Biomass accumulation over 6-day fermentation in R5A liquid media. 25 ml media were inoculated with inoculum directly from glycerol stocks to OD_450_ 0.03. Following incubation, 1 ml culture sample was removed and spun for 3 min at 14,000 rpm. Supernatants were discarded and the pellet was dried at 80 °C overnight and weighed. Values are means of three replicates. Error bars represent standard deviation. *DCW* dried cell weight. Deletion of *pfk*_*SA*_ gene and *crp*_SC_ overexpression has no effect on growth rate relative to wild-type whereas Δ*wbla* mutant accumulates ~ 5 times more biomass than the wild type (p < 0.0001). Statistical significance was calculated with Student’s t-test. **c** Chemical structures of paulomenols/paulomycins compounds. Paulomenol B, calculated m/z of 661.26; paulomenol A, calculated m/z of 675.27; paulomycin B, calculated m/z of 786.25; paulomycin A, calculated m/z of 800.27. Paulic acid moiety conferring the antimicrobial activity of paulomycins is indicated with blue shade

We used SCO5426 as the first gene probe encoding one of the three phosphofructokinases found in the *S. coelicolor* genome that is shown to upregulate actinorhodin through increased carbon flux into the pentose phosphate pathway [20]. Blast analysis identified the orthologue gene in *S. albus* genome that shares 89% nucleotide homology, designated as *pfk*_SA_. The left-side flanking regions of *pfk*_SA_ are conserved in both strains as the gene is located next to a cluster of three genes (phosphate acetyltransferase, acetate kinase and pyruvate kinase) involved in pyruvate metabolism. Similarly, we probed *wblA*_SA_ that showed 87% identity to *wblA*_SC_ and SCO1712_SA_ with 76% sequence homology to SCO1712_SC_. We constructed an in-frame deletion of *pfk*_SA_ using a pUC19-based suicide vector where the ampicillin resistance gene was replaced with oriT-apramycin cassette for transfer and selection in *Streptomyces*. The vector harbors two fragments of ~ 1 kb upstream and downstream flanking regions of *pfk* gene that have been fused together at start and stop codons of the gene by overlapping PCR. The plasmid was conjugated into *S. albus* and double crossover mutants were verified by PCR of Apra^S^ colonies (Fig. 1a, b). Wild type and Δ*pfk*_SA_ showed no difference when they grew on MS plates but Δ*pfk*_SA_ mutant was less sensitive to diamide (Fig. 1c). Diamide is an artificial thiol oxidant that forms protein intramolecular disulfide bonds. The reduction of these toxic disulfide bonds is achieved through the action of thioredoxin/thioredoxin reductase system in the presence of NADPH [39]. Similar to *pfk* deletion in *S. coelicolor*, carbon flux towards pentose phosphate pathway due to *pfk* deletion may result in a higher level of NADPH that makes *S. albus* Δ*pfk* mutant more resistant to diamide oxidant. For generating *wblA*_SA_ deletion mutant, we constructed a fosmid library of *S. albus* J1074 and screened for *wblA* sequences. A single fosmid containing the full-length gene was recovered and ReDirect protocol [40] was used to replace *wblA*_SA_ gene with apramycin marker flanked by FRT sites, which was subsequently removed by FLP recombinase resulting in fosmid with seamless *wblA*_SA_ deletion (Fig. 2a, b). The resulting mutagenized fosmid was conjugated into *S. albus* and exconjugants were PCR-screened for double crossover mutant identification (Fig. 2a, b). Similar to *wblA* deletion phenotypes reported in other *Streptomyces* spp. [13, 41–43], the Δ*wblA*_SA_ mutants failed to sporulate (Fig. 2c). Δ*wblA*_SA_ mutants also accumulated more biomass (~ 5-fold) relative to wild type when they grew in R5A media (Fig. 4b). Multiple attempts to knock out SCO1712_SA_ using either Red/ET recombineering or CRISPR-Cas9 systems were proved fruitless, suggesting that SCO1712_SA_ may have an essential role in *S. albus* growth cycle. In order to combine the positive role of *crp* global regulator on secondary metabolism [27] to the above mutants, we heterologously expressed the *crp* gene from *S. coelicolor* into *S. albus* chromosome under the control of a strong constitutive promoter. The coding region of *crp*_SC_ was PCR-amplified, cloned into pIJ10257 conjugative integrative plasmid downstream of ermE*p and transferred in *S. albus* derivative strains using hygromycin selection (Fig. 3a). The overexpression of *crp*_SC_ gene had no effect on the growth rate of *S. albus* in R5A liquid media (Fig. 4b) but interfered with the sporulation process resulting in a white phenotype relative to wild type when *S. albus* grew on MS solid media (Fig. 3b).

### Profiling of the secondary metabolite content in engineered *S. albus* strains

**Fig. 5:**
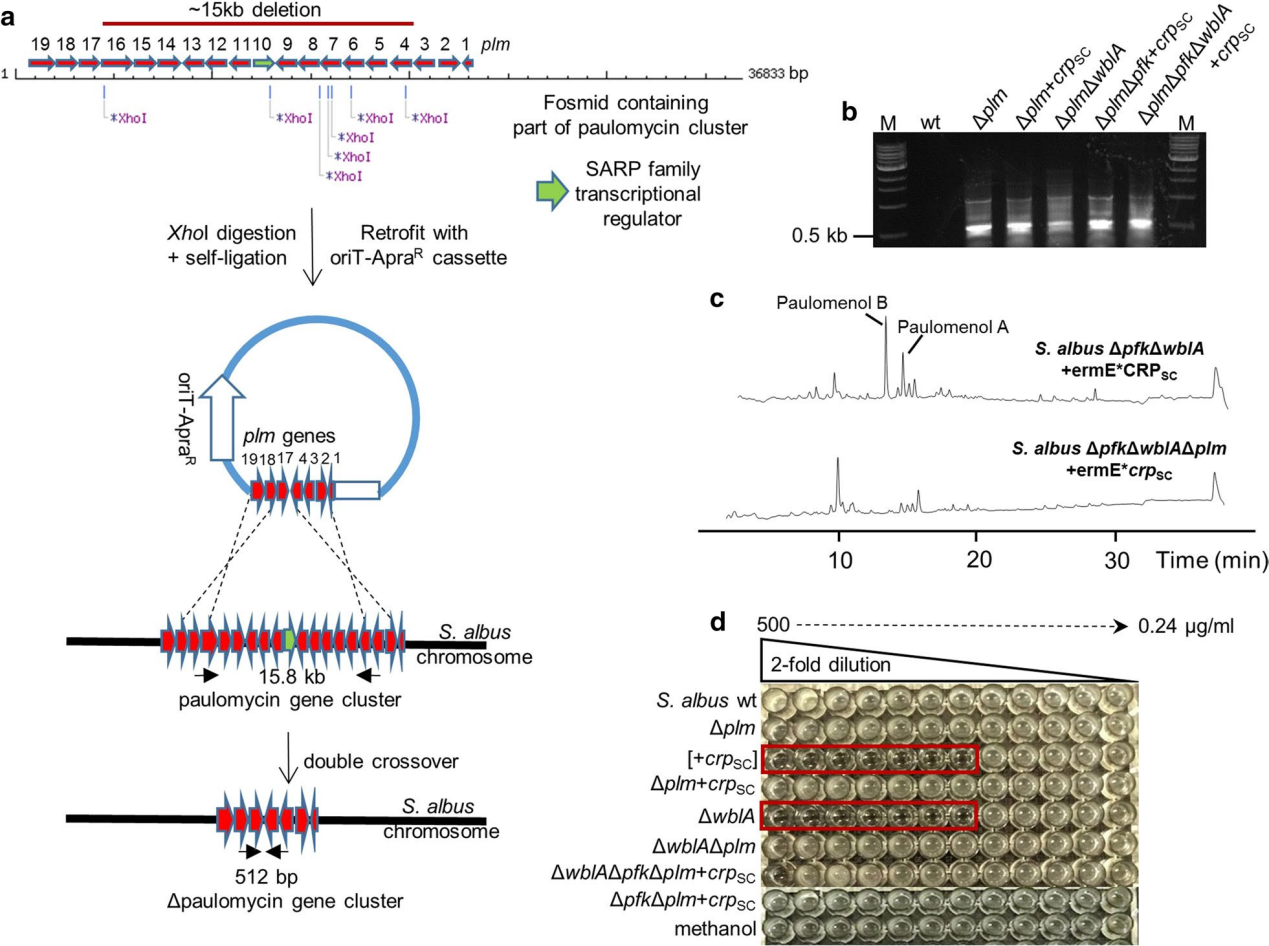
**a** Paulomycin gene cluster knockout strategy. **b** PCR confirmation of 15 kb deletion from the paulomycin gene cluster in different backgrounds. M, 1 kb ladder. Solid black arrows represent primers used for PCR screening and sizes of the PCR products are indicated. Confirmation of upregulation of paulomycin gene cluster **c** in triple mutant *S. albus* Δ*pfk*Δ*wblA*+ermE*crp_SC_ versus *S. albus* Δ*pfk*Δ*wblA*Δ*plm*+ermE*crp_SC_ by LC–MS profiling of culture extracts produced following 6 days of growth in R5A media and **d** antimicrobial assay of culture extracts derived from *S. albus* and engineered strains grown for 4 days in R5A media against *Bacillus cereus*. Minimum inhibition concentration for both *S. albus*+ermE*crp_SC_ and Δ*wblA* derived extracts are reported to be 7.8 µg/ml

Wild type and mutant strains of *S. albus* were grown in R5A media for 6 days and culture extracts were analyzed by LC–MS (Fig. 4a). Although the metabolic profile of Δ*pfk* strain did not differ from that of wild type, Δ*wblA* and [+ermE*p-*crp*_SC_] strains produced a set of metabolites that were absent in wild type extracts. The most dominant peaks (**1**–**4**) appeared at 13 min, 14 min, 20 min and 21 min. The [M + Na] masses of 670, 684, 795 and 809 respectively matched those of paulomenol B/A and paulomycin B/A, respectively. In addition compounds **1**/**2** and **3**/**4** showed absorption spectra with maxima at 322 and 275 nm identical to paulomenols and paulomycin, respectively. In order to genetically verify the production of paulomenols/paulomycins in Δ*wblA* and [+ermE*crp_SC_] strains, we deleted a 15-kb region from the paulomycin (*plm*) gene cluster that includes the pathway-specific regulator *plm*10 (Fig. 5a, b). These corresponding peaks disappeared from the culture extracts of resultant strains in LC–MS analysis (Fig. 5c; Additional file 1: Figure S1). It is difficult to quantitate the production of paulomycins because of their partial degradation to paulomenols. Indeed we observed the transition from paulomycin to paulomenols when analyzing samples daily following inoculation from fermentation broths (Additional file 1: Figure S2). Nonetheless, based on the most dominant paulomenol B peak (peak #1) and the biomass produced by the engineered strains, there was a 2-fold increase in the production rate of the paulomenol B per mg dry biomass in [+ermE*crp_SC_] background relative to Δ*wblA* mutation. Paulomycins differ from paulomenols by the presence of paulic acid that gives the characteristic UV absorption maxima at 275 nm and confers antimicrobial activity against Gram-positive bacteria. Extracts of Δ*wblA* and [+ermE*crp_SC_] strains prepared after 4 days of growth in R5A media were active against *Bacillus cereus* (as low as 7.8 µg/ml) as opposed to extracts derived from wild type and Δ*plm* backgrounds that they showed no antimicrobial activity up to 500 µg/ml tested (Fig. 5d).

Using Regulatory Sequence Analysis Tools (RSAT; http://rsat.eu/) [44], we scanned the paulomycin gene cluster for possible CRP binding sites using the two reported sequence motifs, GTG(N)_6_GNCAC and the one with more relaxed binding specificity GTG(N)_6_GNGAN [27]. The first motif was found within the coding sequence of *plm*12, 28, 29, 35, 37 and *plm*40 genes while the second motif found within the coding sequences of *plm*2, 4, 6–10, 12, 23, 28, 32 and *plm*42 genes as well in the intergenic region *plm*7–*plm*8 genes (Additional file 1: Table S1). Genes *plm*2 and *plm*10 are two of the four transcriptional regulators found in the paulomycin cluster and specifically for *plm*2 gene putative CRP binding site starts five bases upstream of its start codon whereas overlaps the start codon of *plm*42 encoding for dTDP-4-keto-6-deoxyhexose 3,5-epimerase starting nine bases upstream of the corresponding start codon (Additional file 1: Tables S1, S2).

### Heterologous expression of actinorhodin in *S. albus* engineered strains

**Fig. 6:**
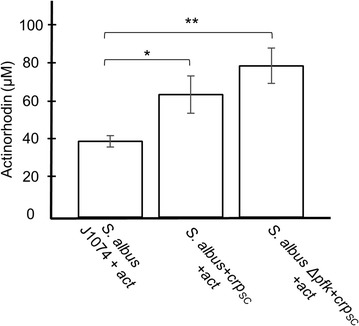
Quantification of the heterologous expression of actinorhodin gene cluster in *S. albus* J1074 and derivative engineered strains. Synergistic effects of *crp*_SC_ overexpression and *pfk*_SA_ deletion on the actinorhodin production were observed. *S. albus*+ermE*crp_SC_ increases the actinorhodin production by 1.6-fold and the double engineered *S. albus* Δ*pfk*+ermE*crp_SC_ strain by 2-fold relative to the *S. albus* J1074 (*p < 0.05, **p < 0.01.). Three different exconjugants from each strain were used in the experiment and values represent means of three biological replicates. Statistical significance was calculated with Student’s t-test and one-way ANOVA from GraphPad Prism 6

Next we wanted to test the effects of the gene-targeted engineering of *S. albus* genome on the expression of heterologous gene clusters. For proof of principle, we used the model actinorhodin gene cluster encoding a diffusible pH-sensing pigment. A single fosmid harboring the gene cluster including flanking regions (SCO5067–SCO5104) was recovered from *S. coelicolor* M145 DNA library and retrofitted with oriT-integrase-apramycin cassette derived from pOJ436 vector. The resulting fosmid was transferred into *S. albus* mutant strains by intergeneric conjugation. The *wblA*_SA_ sporulation-deficient phenotype was not ideal to function as a recipient strain in intergeneric conjugations for routine transfer of foreign gene clusters due to very low transfer rates when using mycelia fragments. Therefore, we restricted the heterologous expression assay on the [ermE*crp_SC_] single mutant and Δ*pfk*+ermE*crp_SC_ double mutant. Increased production of actinorhodin was observed that followed the corresponding sequentially accumulated gene modifications wt < [+ermE*crp_SC_] < Δ*pfk*+ermE*crp_SC_ (Fig. 6). The transcriptional control of *crp*_SC_ gene copy over actinorhodin gene cluster expression in *S. coelicolor* has already been established [27]. In the *S. albus* genetic context, overexpression of *crp*_SC_ gene improved the heterologous expression of actinorhodin by 1.6-fold followed by an additional 1.2-fold when combined with the *pfk*_SA_ deletion, indicating the approximately additive effect of these mutations to the actinorhodin biosynthesis.

## Discussion

The biosynthesis of natural products is highly regulated and gene clusters often remain silent until suitable conditions are met. The expression of secondary metabolite pathways is under the control of tight and complex hierarchical regulatory networks that integrate multiple nutritional and environmental signals perceived by pleiotropic and pathway-specific transcriptional regulators. Several successful strategies have been employed in the past, targeting both groups of regulators for activation of silent pathways in many *Streptomyces* species. *S. albus* J1074 is a gifted strain for heterologous expression of secondary metabolite biosynthetic genes of broad (meta)genomic origin. Given the current plethora of sequence availability for many diverse biosynthetic gene clusters, further improvement of this strain utilizing not only pathway-specific mutagenesis but broad host engineering approaches could result in a promising drug discovery platform.

Recent studies have linked oxidative stress with antibiotic production in *Streptomyces coelicolor* [45–47] where secondary metabolite production could function as a homeostatic response to overactive cellular oxidative phosphorylation. Elevated levels of NADPH and ATP, induction of disulfide stress-responsive genes are all accompanied with the onset of antibiotic production. Phosphofructokinase (pfk) functions at the branching point between glycolysis and pentose phosphate pathway (PPP). Deletion of its gene in *S. coelicolor* confers more resistance to the thiol oxidant diamide than the wild-type strain and results in overexpression of actinorhodin by diverting fructose-6-phosphate into PPP leading to increased levels of NADPH cofactor that is necessary for the function of many biosynthetic redox enzymes [20]. Avermectin production is correlated with increased activity of pentose phosphate pathway in *S. avermetilis* [48]. *S. hygroscopicus* Δ*pfk* mutant increases rapamycin production by 30.8% [21]. Here we showed that *S. albus* Δ*pfk* strain is more resistant to diamide than the wild type. Therefore, *S. albus* Δ*pfk* strain more likely overexpresses the pentose phosphate pathway resulting in higher intracellular NADPH availability that allows the mutant to cope more efficiently with the oxidative stress than the wild-type. The increased supply of the NADPH cofactor allows this heterologous host to improve the production of herbicidal thaxtomins, whose biosynthesis relies on the NADPH-dependent function of cytochrome P450 enzymes (unpublished data).

WblA, member of the WhiB-like proteins, has also a negative effect on disulfide stress response [49]. This family of proteins contains a [Fe-S] structural element that converts them into redox sensors. One of the key characteristics of *wblA* gene deletion in *S. coelicolor* is the limited sporulation and prolonged fast growth [13]. WblA is shown to be involved in downregulation of antibiotic production. In fact, deletion of *wblA* homologue genes in *S. ghanaensis*, *S. peucetius*, *S. somaliensis*, *S. venezuelae*, *S. ansochromogenes* resulted in the overexpression of moenomycin, doxorubicin, violapyrone B, pikromycin and tylosin analogs biosynthetic genes respectively, suggesting that *wblA* functions as a down-regulator for secondary metabolites biosynthesis in *Streptomyces* species [39, 40, 50–52]. AdpA, another global regulator of secondary metabolism, is shown to repress the expression of *wblA* in *S. coelicolor* indicating that the two regulators are members of the same regulon [15]. Our results indicate that deletion of *wblA* in *S. albus* blocked completely the sporulation process and the Δ*wblA* strain overproduced paulomycins A/B during fermentation, which finally ended in the accumulation of paulomenols A/B after 6 days of growth. The increased biomass observed in the *S. albus* Δ*wblA* mutant may also contribute to the production enhancement of paulomycins previously undetected by analytical techniques. The induced production of paulomycins may be a cellular response to utilize the increased energy levels generated during the extended fast growth of this mutant, which is not favorable for the cell during nutrient limitation.

cAMP receptor protein (CRP) has been extensively studied in *E. coli* for its involvement in carbon catabolite repression. In *Streptomyces*, deletion of the gene results in defects in germination and sporulation [53]. Exogenous addition of cAMP in *S. coelicolor* stimulated actinorhodin production [54] and it has been shown that CRP protein recognizes its cognate binding sites in 8 out of the 22 biosynthetic gene clusters in *S. coelicolor* genome affecting their gene expression, as well as promoter region of acetyl-CoA carboxylase that generates malonyl-CoA precursor for secondary metabolism [27]. CRP-transcriptionally targeted genes are also cross-modulated by two component regulatory cascades such as *PhoR*–*PhoP* and *AfsK*–*AfsR*–*AfsS* that link phosphate homeostasis and antibiotic production in *Streptomyces* [27]. Overexpression of *crp*_SC_ gene in *S. albus* produced a white phenotype and induced the production of paulomycin-related metabolites with actually higher specific rate than the Δ*wblA* strain. Putative CRPsc binding sites are detected in the paulomycin cluster providing a genetic framework for the modulation of paulomycin gene expression by this global regulator.

Heterologous expression is an efficient way to discover new metabolites when the original producer is not known or is not genetically amenable. While Δ*wblA* non-sporulating phenotype does not allow the efficient transfer and heterologous expression of large gene clusters, here we demonstrated that *pfk*_SA_ gene deletion (even though it does not have any apparent effect on the *S. albus* native metabolome) plays a positive role in the heterologous expression of actinorhodin and acts synergistically with *crp*_SC_ gene, yielding a total ~ 2-fold increase in actinorhodin production relative to the wild-type *S. albus* expressing the *act* genes. *S. albus* has a recorded ability to heterologously express other type II polyketides [55]. Nae et al. [56] have constructed a *S. coelicolor* Δ*pfk*Δ*wblA*ΔSCO1712 triple mutant that also showed a synergistic effect in stimulating actinorhodin and other type II polyketide biosynthesis [57]. We attempted to delete SCO1712 homologue gene in *S. albus* but our efforts were unsuccessful using both recombineering (REDIRECT method) and CRISPR/Cas9 approaches. Interestingly, we were able to obtain single cross-over mutants using REDIRECT method, potentially suggesting that complete elimination of the gene through a double cross-over event is not possible due to the essential role of SCO1712_SA_ in *S. albus* growth. Deletion of *SCO1712* in *S. coelicolor* results in overproduction of actinorhodin and Red antibiotics and affects morphological differentiation by earlier formation of aerial mycelium and sporulation deficient phenotypes. However, this gene is dispensable for growth in *S. coelicolor* [36]. Given the natural ability of *S. albus* to express type II PKS genes, the constructed strains with cleaner background described here like Δ*pfk*Δ*plm*+ermE*crp_SC_ will be proved useful tools for the characterization of novel type II polyketides.

## Conclusions

**Fig. 7:**
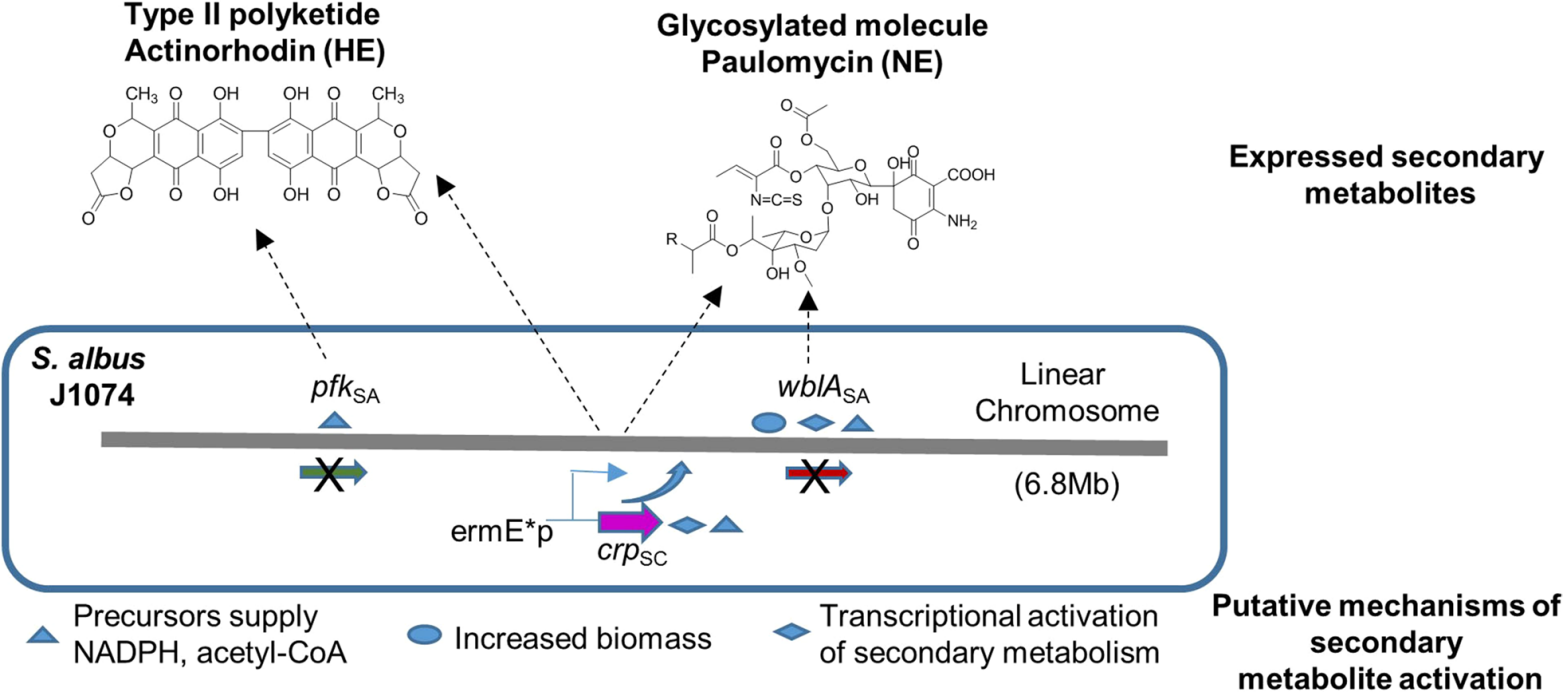
Host engineering overview for *S. albus* J1074 strain improvement that allows the expression of silent native biosynthetic pathways and the enhancement of the heterologous expression of foreign gene clusters. *HE* heterologous expression, *NE* native expression

Gene-targeted engineering of *S. albus* J1074 genome resulted in improved gene expression capabilities of secondary metabolism. Deletion of *pfk* gene supplied increased levels of NADPH reducing cofactor to the biosynthetic pathways containing NADPH-dependent enzymatic steps. Heterologous expression of actinorhodin was assisted by this genetic modification. Overexpression of the transcriptional regulator CRP from *S. coelicolor* in the *S. albus* background activated the expression of paulomycins and function synergistically with global regulators controlling other modes of regulation of secondary metabolism like pfk for the heterologous expression of actinorhodin. Deletion of the global antibiotic down regulator WblA, induced the production of paulomycins in response to prolong fast growth and biomass accumulation. Overall we showed that rational, multiplex genome engineering (Fig. 7) is an efficient way to unlock the expression of native metabolites and further enhance the heterologous expression properties of gifted hosts.

## Additional file

**Additional file 1.** Figures S1–S6 and Tables S1, S2.

## Abbreviations

NADPH: nicotinamide adenine dinucleotide phosphate (reduced form)
ATP: adenosine triphosphate
MOPS: 4-morpholinepropanesulfonic acid
TE: tris-EDTA
EDTA: ethylene diamine tetraacetic acid
PKS: polyketide synthase
Pfk: phosphofructokinase
wblA: whiB-like gene A
CRP: cAMP receptor protein
