# Supplementary material for: Rational engineering of Streptomyces albus J1074 for the overexpression of secondary metabolite gene clusters
Source: Microb Cell Fact. 2018 Feb 17;17:25. doi: 10.1186/s12934-018-0874-2 (PMC5816538; doi:10.1186/s12934-018-0874-2)
Supplement: Supplementary file 1 — Additional file 1. Figures S1–S6 and Tables S1, S2. [file 12934_2018_874_MOESM1_ESM.pdf]

Additional file for:

Title: Rational engineering of *Streptomyces albus* J1074 for the overexpression of secondary metabolite gene clusters

Authors: Dimitris Kallifidas[1,2*], Guangde Jiang[1,2], Yousong Ding[1,2], Hendrik Luesch[1,2*]

1 Department of Medicinal Chemistry, College of Pharmacy, University of Florida, Gainesville, Florida 32610, USA
2 Center for Natural Products, Drug Discovery and Development (CNPD3), University of Florida, Gainesville, Florida 32610, USA
*Corresponding authors: dkallifidas@ufl.edu, luesch@cop.ufl.edu

Figure S1: Confirmation of upregulation of paulomycin gene cluster by CRP_{SC} overexpression and $WblA_{SA}$ deletion using LC-MS profiling. **Page S2**
Figure S2: Monitoring of the paulomycin A/B and paulomenol A/B production by LC-MS profiling. **Page S3**
Figure S3: MS fragmentation pattern of paulomenol A **Page S4**
Figure S4: MS fragmentation pattern of paulomenol B **Page S5**
Figure S5: MS fragmentation pattern of paulomycin A **Page S6**
Figure S6: MS fragmentation pattern of paulomycin B **Page S7**

Table S1: Detection of putative CRP_{SC} binding sites in paulomycin gene cluster using $GTG(N)_6GNCAC$ motif. **Page S8**
Table S2: Detection of putative CRP_{SC} binding sites in paulomycin gene cluster using $GTG(N)_6GNGAN$ motif. **Page S8**

Figure S1: Confirmation of upregulation of paulomycin gene cluster (*plm*) by CRP$_{SC}$ overexpression and WblA$_{SA}$ deletion by LC-MS analysis of organic extracts prepared from *Streptomyces albus* J1074 engineered strains grown in R5A media for 4 days. 1, paulomycin B; 2, paulomycin A; 3, paulomenol B; 4, paulomenol A.

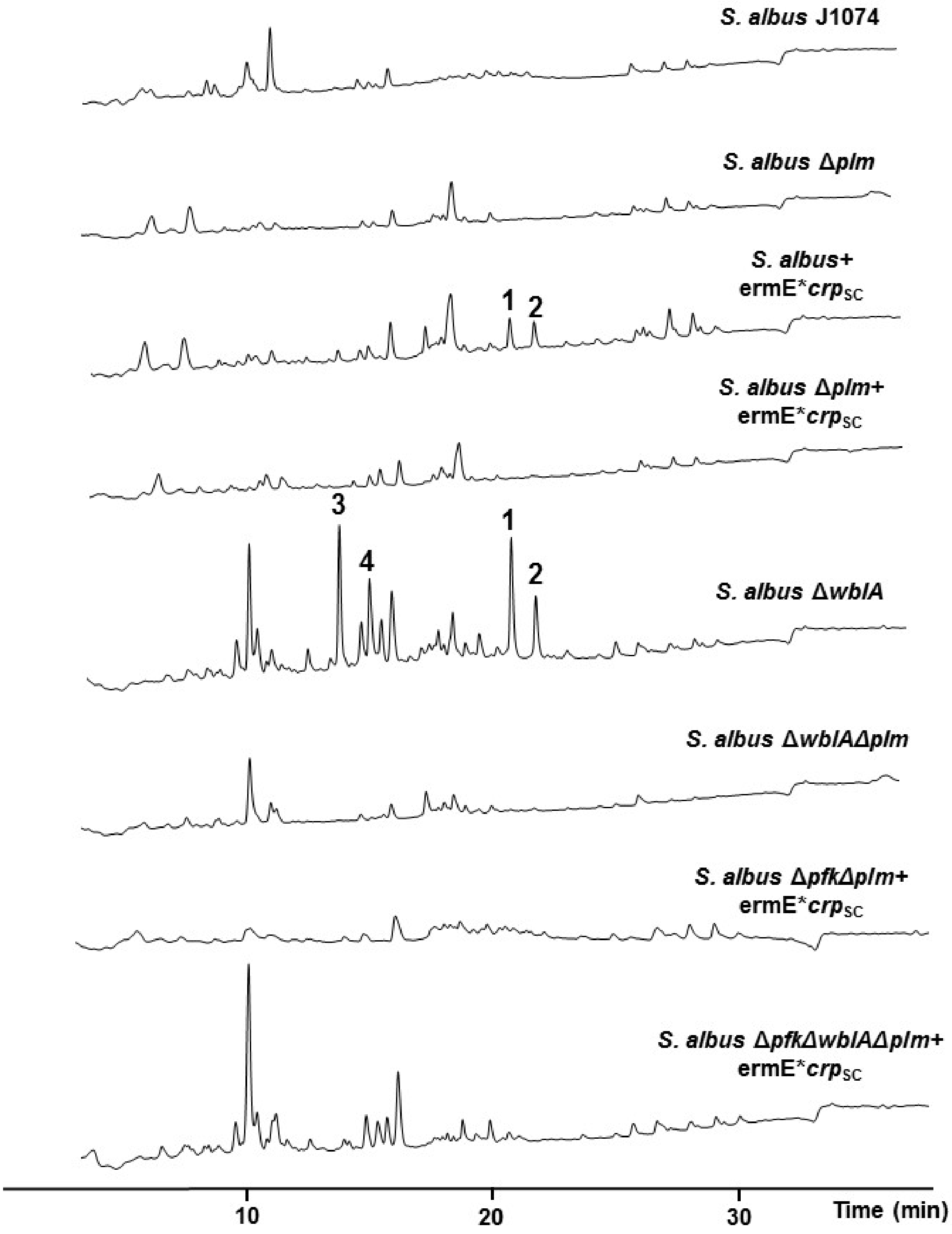

Figure S2: LC-MS analysis of *S. albus* Δ*wblA* culture extracts collected daily for 6 days from R5A fermentation broths. 1, paulomycin B; 2, paulomycin A; 3, paulomenol B; 4, paulomenol A.

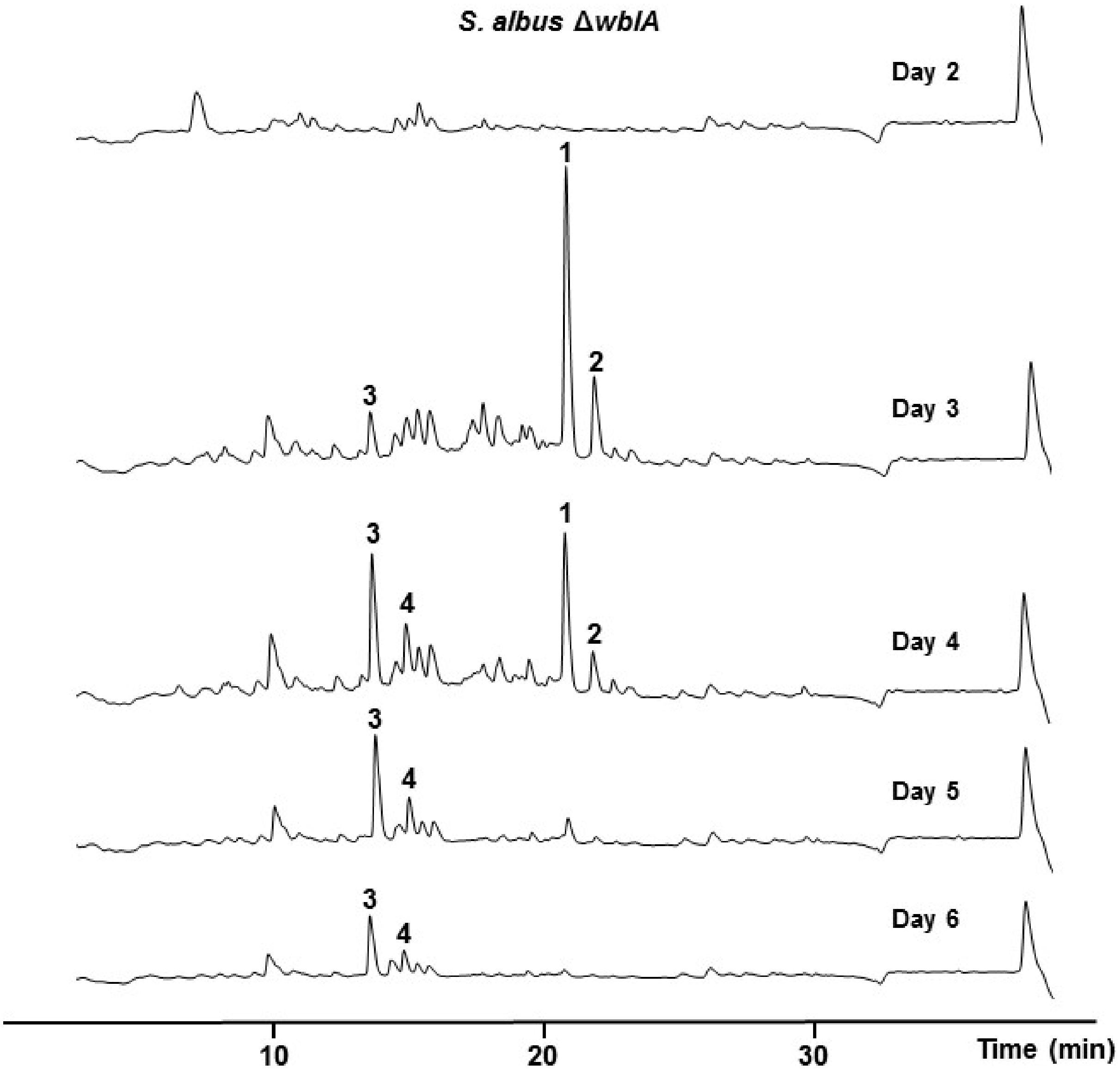

Figure S3: MS fragmentation pattern of paulomenol A. Negative mode.

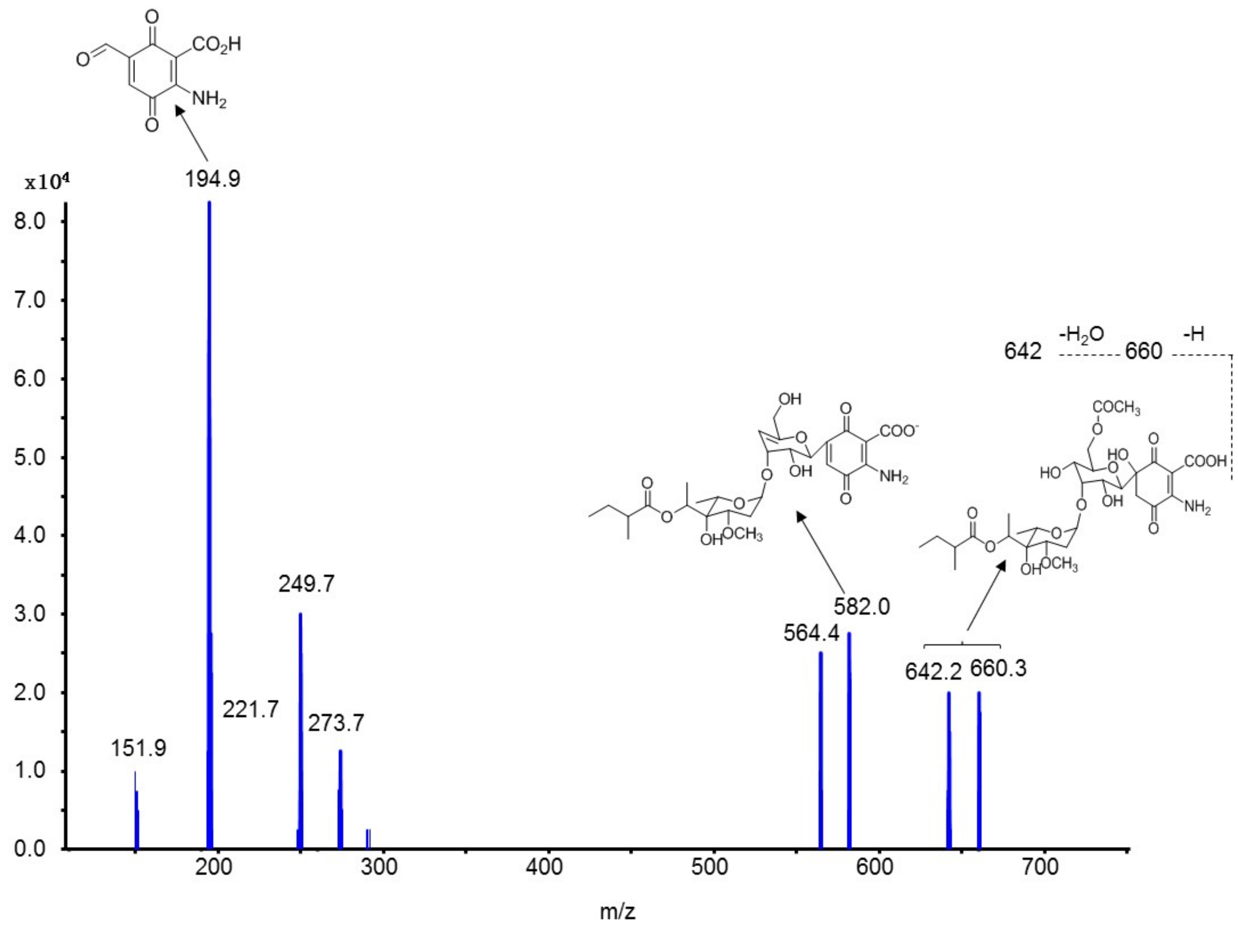

Figure S4: MS fragmentation pattern of paulomenol B. Negative mode.

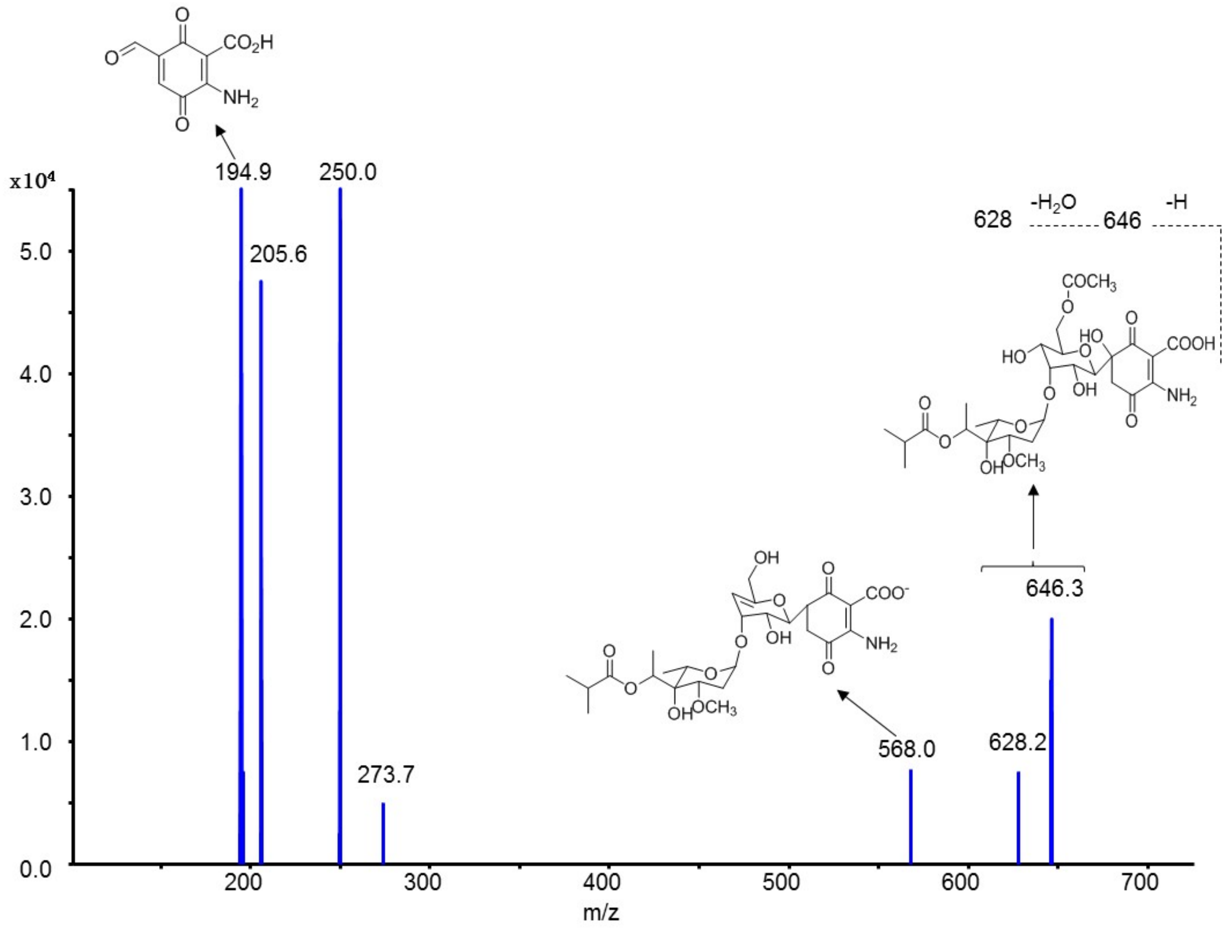

Figure S5: MS fragmentation pattern of paulomycin A. Negative mode.

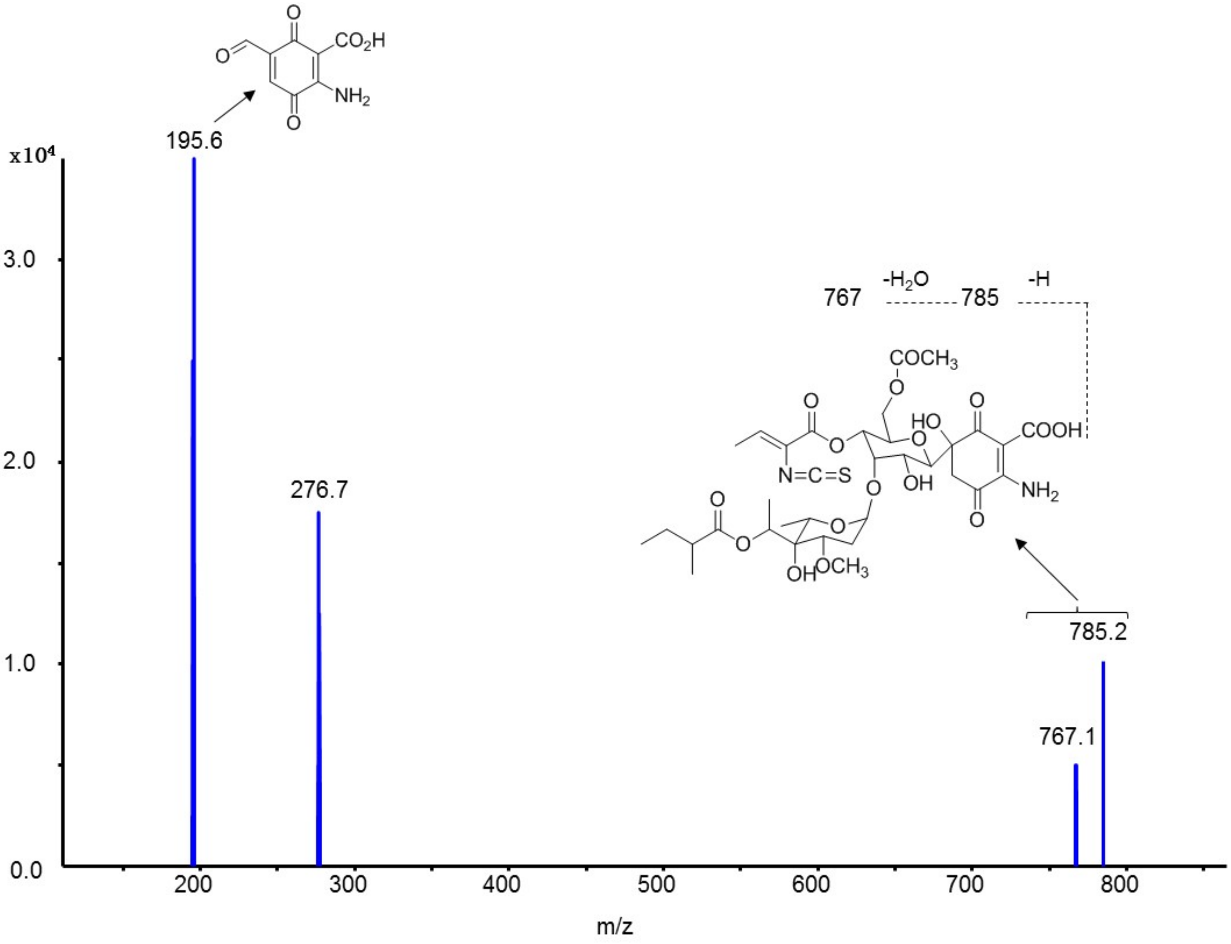

Figure S6: MS fragmentation pattern of paulomycin B. Negative mode.

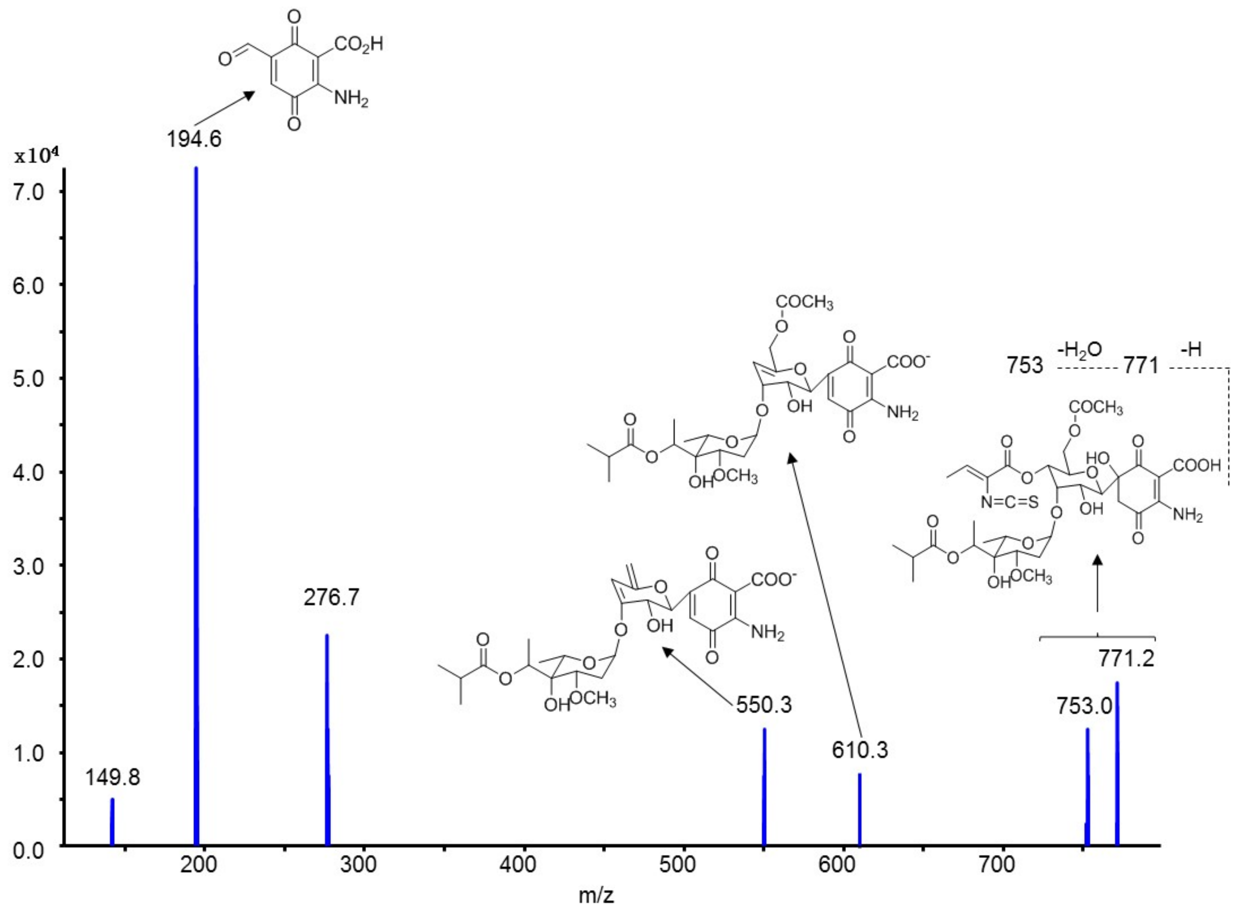

Table S1: Sequence scanning of paulomycin (*plm*) gene cluster for the detection of putative CRP$_{SC}$ binding sites using the GTG(N)$_6$GNCAC motif. Distance from the beginning of the motif to the start codon of the corresponding genes is reported. Negative values indicate sites upstream of start codons while positive values indicate sites within open reading frames.

***plm* gene**	**Function**	**distance**	**Binding site**
plm 12	Glycosyltransferase	+130	cctcGTGGACACCGCCACcggc
plm 28	Putative sulfotransferase	+628	ggcgGTGGCCATGGCCACggag
plm 29	Aminotransferase	+91	cctgGTGCCGCTCGTCACcggc
plm 35	Ribulose-5-phosphate-4-epimerase	+29	acgcGTGAGCGAGGGCACcccg
plm 37	Acyl-CoA dehydrogenase	+763	cgcgGTGGGGCTCGCCACcgcg
plm 40	dTDP-4-keto-6-deoxy-L-hexose 2,3-reductase	+301	ccggGTGGTGCTGGCCACcaag

Table S2: Sequence scanning of paulomycin (*plm*) gene cluster for the detection of putative CRP$_{SC}$ binding sites using the GTG(N)$_6$GNGAN motif. Distance from the beginning of the motif to the start codon of the corresponding genes is reported. Negative values indicate sites upstream of start codons while positive values indicate sites within open reading frames.

***plm* gene**	**Function**	**distance**	**Binding site**
plm 2	TetR-family transcriptional regulator	-5	ggggGTGCGATGCGGGACgcgc
plm 4	Oxidoreductase	+162	ttcgGTGGGGACGGGGACacgg
plm 6	EmrB/QacA subfamily transporter	+1230	cagcGTGCCCGCCGCGACcacg
plm 7	Elongation factor G1	+1620	gttcGTGAACAAGGTGACcggt
plm 8	Dehydrogenase E1 alpha subunit	-299	gggaGTGACCGACGCGACagcg
*plm*8	Dehydrogenase E1 alpha subunit	+900	gctgGTGGCGGAGGCGAGggac
plm 9	Dehydrogenase E1 beta subunit	+333	cggcGTGCCCGTGGTGACccgg
plm 9	Dehydrogenase E1 beta subunit	+507	ggtgGTGCTCATCGAGAAccgc
plm 9	Dehydrogenase E1 beta subunit	+825	cgcgGTGGTGGCCGAGAAcgta
plm 9	Dehydrogenase E1 beta subunit	+864	cccgGTGCGGCGGGTGACcctg
*plm*10	SARP-family transcriptional regulator	+153	ccagGTGCTCCCGGCGACaacg
plm 12	Glycosyltransferase	+672	ggacGTGGCCGGGGAGACgctc
plm 23	C-glycosyltransferase	+204	gggcGTGCCCCTGGTGAGgtcc
plm 28	Putative sulfotransferase	+390	cctcGTGGTGTGCGCGAGcgag
plm 32	Acyl-CoA synthase	+1221	cgtgGTGCTCGAAGTGACcgac
plm 42	dTDP-4-keto-6-deoxyhexose 3,5-epimerase	-9	tgaaGTGGGGAGAGTGAGcccc
